# Adaptive Temporal Matched Filtering for Noise Suppression in Fiber Optic Distributed Acoustic Sensing

**DOI:** 10.3390/s17061288

**Published:** 2017-06-05

**Authors:** İbrahim Ölçer, Ahmet Öncü

**Affiliations:** 1 TÜBİTAK BİLGEM, Barış Mah., Dr. Zeki Acar Cad., Gebze 41470, Kocaeli, Turkey; 2 Electrical & Electronics Engineering Department, Boğaziçi University, Bebek, İstanbul 34342, Turkey; ahmet.oncu@boun.edu.tr

**Keywords:** fiber optic sensors, Rayleigh scattering, vibration detection, adaptive temporal filtering, matched filters, Wiener filters, structural health monitoring

## Abstract

Distributed vibration sensing based on phase-sensitive optical time domain reflectometry (ϕ-OTDR) is being widely used in several applications. However, one of the main challenges in coherent detection-based ϕ-OTDR systems is the fading noise, which impacts the detection performance. In addition, typical signal averaging and differentiating techniques are not suitable for detecting high frequency events. This paper presents a new approach for reducing the effect of fading noise in fiber optic distributed acoustic vibration sensing systems without any impact on the frequency response of the detection system. The method is based on temporal adaptive processing of ϕ-OTDR signals. The fundamental theory underlying the algorithm, which is based on signal-to-noise ratio (SNR) maximization, is presented, and the efficacy of our algorithm is demonstrated with laboratory experiments and field tests. With the proposed digital processing technique, the results show that more than 10 dB of SNR values can be achieved without any reduction in the system bandwidth and without using additional optical amplifier stages in the hardware. We believe that our proposed adaptive processing approach can be effectively used to develop fiber optic-based distributed acoustic vibration sensing systems.

## 1. Introduction

Distributed fiber optic sensor systems had been introduced decades ago and are attracting growing interest as a practical and efficient means for several vibration detection applications. The technology is becoming one of the core technologies in many areas such as structural health monitoring applications, mechanical processes and transportation systems [1,2,3]. Combined with appropriate instrumentation techniques, the fiber optic cable itself acts as a sensor to highlight physical changes and disturbance information in various structures as a monitoring probe. Due to the intrinsic properties of fiber optic cables, various types of distributed fiber optic sensors have been introduced and demonstrated for monitoring different parameters at any point along a fiber. Phase-sensitive optical time domain reflectometry (ϕ-OTDR), which is based on the analysis of the Rayleigh backscattered optical power in the fiber optic cables, is one of these types. This technology has been demonstrated as a cost-effective and ideal way for the distributed measurement of acoustic vibrations, which may be due to different mechanical sources surrounding the fiber optic cable. Crack detection of materials in civil structures and anomaly detections of engines for disaster prevention can be listed as the most common application areas of this technology. Monitoring oil pipelines for chemical leakage, railroad safety and intrusion detection for perimeter security applications are gaining popularity for distributed acoustic vibration sensing. In most of these distributed acoustic sensing (DAS) applications, the frequency range of the acoustic events of interest, such as the crack detection, could be as high as several kHz. For this reason, a vibration sensor with a high frequency response range is desired. Although there is growing interest for this type of fiber sensor, very limited research on the signal processing of the Rayleigh backscattered waveform has been reported. The conventional methods for performance improvement mostly rely on coherent summation of *N* successive ϕ-OTDR traces and taking the average of them to improve the SNR by $\sqrt{N}$. The main drawback of this classical approach is the inherent reduction of system bandwidth by a factor *N*; thus, this plain averaging approach is not suitable to detect high frequency acoustic vibrations [4,5]. Another disadvantage stems from the stochastic nature of optical Rayleigh backscattering: the received ϕ-OTDR traces are always noisy, and the highly fluctuating amplitude profile of ϕ-OTDR traces makes it difficult to reveal vibration information [6,7,8].

There exist several alternatives proposed in the literature to enhance the DAS system sensitivity. One of them is called heterodyning, which is based on optical mixing of narrow linewidth light source power with the backscattered weak Rayleigh signal energy before the photodetector [9]. Although appropriate mixing of the source power with the backscatter signal enhances the detection, the change of the relative state of polarization (SOP) between the interfering optical fields is one of the main problems encountered for this detection scheme. The randomly-varying phase difference between the source and backscattered energy may even result in a zero current at the photodetector output. This instability issue can be managed if polarization diversity detection techniques [10,11] are used or if the SOP is kept constant by using all polarization maintaining (PM) components in the setup including the sensing fiber [12]. Note that in this case, the system cost increases considerably with the usage of all PM components. In addition to the aforementioned methods, bandpass filters and optical amplifiers are introduced in the system to enhance the SNR to a certain degree. In one alternative application, moving average and moving differential methods are employed to reduce the noise floor; vibration events with the highest detectable frequency around 2.25 kHz and a spatial resolution of 2 m are reported [9]. Both the separate averaging method (SAM) and the moving average and differential methods suffer from the inherent low bandwidth of the averaging technique. According to the well-known Nyquist theorem, the maximum detectable frequency (MDF) is half of the sampling frequency (i.e., pulse repetition frequency (PRF)/2 Hz). When using SAM with an averaging size of *M*, the MDF is reduced to PRF/2*M*, while MDF is only increased at most twice this value with the moving average method (i.e., MDF = PRF/*M*) [9]. A different technique based on edge detection and two-dimensional processing of backscattered optical traces can increase the SNR to 8.5 dB compared to these conventional methods [13] .

Besides the conventional time-domain techniques mentioned above, transform domain methods are used as alternatives for SNR improvement. By Fourier transforming the Rayleigh backscattering traces, 9.5 dB of SNR was achieved with a spatial resolution of 3.7 m [14]. One method used a wavelet technique for noise reduction with resulting increases in both the highest detectable frequency and a spatial resolution of 8 kHz and 0.5 m, respectively [15]. In the wavelet denoising method used in [15], after computation of the discrete wavelet transform (DWT) for the raw ϕ-OTDR traces, an appropriate selection of the threshold is important to disregard the wavelet coefficients associated with the noise components. After this partial removal of the wavelet coefficient, the signal is recovered by the inverse DWT computation. Since the noise components usually correspond to the small valued wavelet coefficients, weak high frequency components very close to the noise level may be missed in this technique. A comprehensive summary of the state-of-the-art for ϕ-OTDR systems is provided in [16].

None of the above-mentioned methods are convenient to detect weak vibrations that are very close to the noise level. Additionally, all system configurations presented in these references [9,10,11,12,13,14,15] employ at least one extra component for amplification, usually an erbium-doped fiber amplifier (EDFA) in the transmission stage to increase the SNR of the ϕ-OTDR system, which increases the system costs.

Weak signal detection in noisy environments has always been a cumbersome problem in diverse areas of signal processing. Adaptive filtering is a heavily-used signal processing technique to detect specifically weak signals under harsh conditions. The adaptive filter theory has found numerous applications in the radar, sonar and speech processing fields, and it is well documented in the literature [17,18]. In our previous work, we demonstrated the efficacy of such an adaptive filtering approach with simulated ϕ-OTDR signals and obtained promising results in the detection of weak acoustic perturbations [19]. Those adaptive filtering simulations were based on the classical Wiener approach and the computation of the adaptive filter coefficients was based on the minimization of a cost function called the mean square error (MSE). In the minimum mean squared error (MMSE) approach, the error term to be minimized is actually the squared difference between the estimated signal and the original signal. This minimization is carried out by incorporating the dynamics of the noise environment. Under stationarity and Gaussian noise assumptions, the computed Wiener coefficients lead to the optimum detection performance [17,18]. However, due to the statistics of ϕ-OTDR signals and noise, the optimality conditions is not met in a generic DAS application. On the other hand, even with its sub-optimal detection performance, the MMSE approach has been successfully demonstrated as a good candidate for vibration detection in DAS applications. Thus, these promising results have motivated us to study the adaptive filtering approach more deeply for relevant fiber optic sensing applications.

Briefly speaking, the final Rayleigh backscattered signal in ϕ-OTDR systems is fairly weak, and careful processing must be employed to improve the signal quality. As stated above, optical amplification is usually required for higher SNR levels. For coherent detection-based ϕ-OTDR, there is an inherent optical amplification achieved by carrying the weak backscatter signal onto a relatively much stronger reference signal. However, due to the stochastic nature of Rayleigh scattering, the amplitude fluctuations of the scattered optical traces form a speckle-like profile, which can mask the phase modulation of the perturbation. The above-mentioned problems are the main motivating facts for us to seek robust techniques to improve the vibration detection capability of DAS systems. For this purpose, we extend our adaptive filtering approach in two aspects in this paper: first, an experimental coherent detection-based ϕ-OTDR system has been developed to handle real signals. Secondly, instead of the aforementioned MMSE approach, we now propose to apply an adaptive matched filtering approach for maximizing the SNR in DAS applications. While the MMSE approach seems to be popular in statistical inference and communications signal processing, the maximum SNR (MSNR) criterion is the most popular method in radar and general detection theory. It has been proven theoretically that the MMSE and MSNR estimators are in fact equivalent even when the noisy observations are non-Gaussian [20]. Thus, by definition, the MSNR method seems to be the most appropriate approach for vibration detection, and we turned our attention to the classical matched filter theory for the adaptation of the MSNR approach in DAS applications.

In the research study reported here, neither booster nor pre-amplifier optical stages are used the system to improve the SNR. A simple coherent detection ϕ-OTDR system has been developed and used without any optical amplification. The experiments conducted in the laboratory with fiber on reels showed that the adaptive temporal filtering (AMF) method can be used to detect high frequency components close to the Nyquist limit without using the conventional trace averaging and denoising techniques. Additional field test results demonstrated the capability of the same AMF method to detect low frequency vibrations, as well, from a buried fiber optic cable with a lateral sensitivity of 25 m. To our best knowledge, this is the first time that an AMF approach is experimentally tested for noise reduction and to enhance the vibration detection performance in distributed fiber optic acoustic sensing. The experimental results show that the temporal AMF technique has a great potential to be used for DAS applications, especially for structural health monitoring (SHM), where high frequency responses are desired.

Our paper is organized as follows: Section 2 is devoted to the detailed description of the proposed approach. First, the ϕ-OTDR signal model used is presented, and then, the intuition behind the AMF method is described. Results of laboratory experiments and field tests are given in Section 3 with discussions. Finally, the last section concludes with comments and proposals for a future study.

## 2. Adaptive Matched Filtering Approach for ϕ-OTDR Systems

### 2.1. The Signal Model

**Figure 1:**
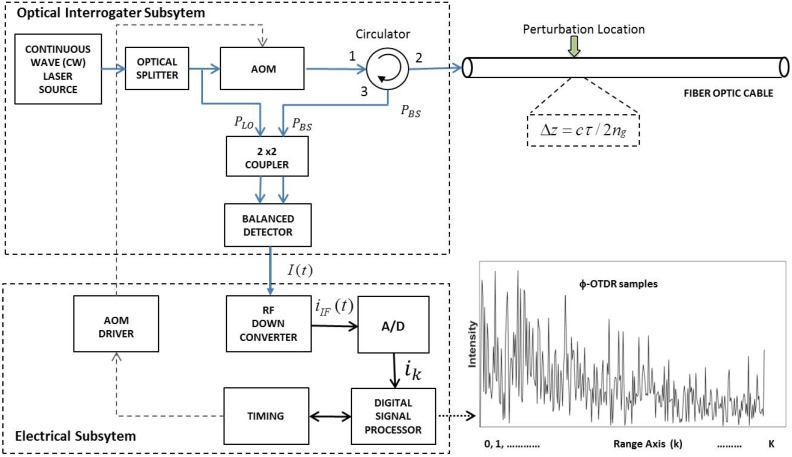
A coherent detection-based generic phase-sensitive optical time domain reflectometry (ϕ-OTDR) architecture.

In this paper, our focus is on a single coherent detection-based ϕ-OTDR system as shown in Figure 1. The fundamentals of coherent detection-based ϕ-OTDR systems are well known and documented [1]. They are advantageous over direct detection systems due to the inherent optical amplification employed by the mixing of the local oscillator (LO) power ($P_{LO}$) with the weak Rayleigh backscattered power $P_{bs}$, in which $P_{LO}$ is directly obtained from the laser source by an optical splitter as shown in Figure 1.

With the assumption that the 180 degree phase shift is preserved between the arms of the coupler, a balanced detector with two perfectly matched arms will eliminate the direct current (DC) and common mode terms. Following this further SNR improvement at the optical-electrical conversion stage, as the intermediate frequency Δω is introduced by the acousto-optic modulator (AOM), the electrical signal is down-converted to baseband with an electrical LO (ELO) equal to the AOM frequency. After appropriate filtering, the analog baseband signal can be written as:

(1) $$i\left(t\right)\propto \sqrt{P_{\mathrm{LO}}P_{\mathrm{BS}}}\cos\left(\theta \left(t\right)\right)$$

where θ(t) denotes the relative polarization angle between the LO signal and the backscattered signal. This cosine term is simply the representative of the SOP effect. The in-phase and quadrature (I/Q) direct down-conversion stage is followed by A/D converters for digitization and further digital processing stages. The complete chain of electrical signal processing blocks can form an ensemble of a single electrical subsystem as depicted in Figure 1.

The ϕ-OTDR system temporally samples the signal environment by transmitting a coherent burst of *N* pulses of width τ at a constant pulse repetition frequency (PRF), $f_{r}=1/T_{r}$, where $T_{r}$ is the pulse repetition interval (PRI).

**Figure 2:**
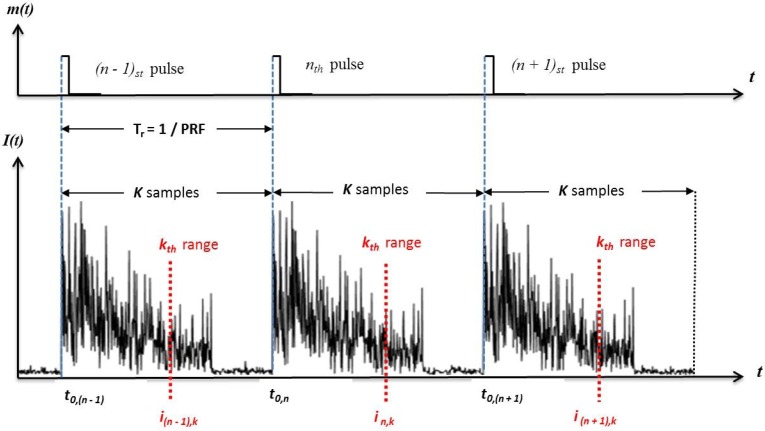
Demonstration of fast and slow-time axes on ϕ-OTDR traces.

As shown in Figure 2, the ϕ-OTDR system collects *K* samples between these successive pulses. These collected optical returns are all representative of virtual sensor outputs along the fiber’s range extent, and they are referred to as range bins. The spatial length of these range bins is called the “spatial resolution (SR)” of the system and coupled to the optical pulse width via Δz=c$\tau /2n_{g}$, where c is the speed of light in a vacuum and $n_{g}$ is the group refractive index. To form a “temporal”snapshot from these optical returns, let us consider the returns for a specific range bin *k*-th range bin, which can be represented by an *N×1* column vector as:

(2) $$I_{k}={\left[\begin{array}{cccc} i_{1,k} & i_{2,k} & ... & i_{N,k} \end{array}\right]}^{T}$$

the scalar $i_{n,k}$ , n∈[1,N],k∈[1,K] represents the final baseband signal detected for the *n*-th pulse at the *k*-th range bin. The superscript *T* denotes the transpose operation. Vector and matrix notations are presented by boldface italic characters throughout the text.

These samples illustrated by dashed red lines in Figure 2 are collected on the “slow-time axis” and should not be confused with the ϕ-OTDR traces, which are plots of returned optical power versus time. In contrast, the sampling interval between the range bins is governed by the sampling rate of the analog-to-digital (A/D) converter, and these samples are collected on the “fast-time axis”. We will use a term called the temporal processing interval (TPI) to refer the time interval over which the *N* pulse returns are collected. Thus, the TPI length of the ϕ-OTDR system processor is $NT_{r}$ s. With a post-detection ϕ-OTDR bandwidth *B* (Hz) selected (i.e., B=1/τ), the sequential processing of the TPI frames is executed by the system as follows: at the output of the balanced detector, the electrical intermediate frequency (IF) signal is down-converted to baseband with an ELO signal frequency equal to the one introduced by the AOM frequency. The down-converted signal is then low-pass filtered and digitized with a sampling frequency at least twice the bandwidth. Digital decimation may be required to reduce the number of I/Q (i.e., in-phase and quadrature) samples per range bin to one. At the final stage, each TPI frame consists of exactly *KN* samples, which are stored in memory for subsequent digital signal processing (DSP) algorithms.

In this research, our primary concern is the sequential processing of every range bin along the fiber under test (FUT) axis. Our goal is to make a decision whether there is a vibration or not at a specific location. In general, along the FUT axis, it is worth recognizing that the detected signal will consist of two main components:

(3) $$H_{0}:I=I_{\mathrm{RB}}+I_{\mathrm{rec}}$$

The condition $H_{0}$ is the null-hypothesis or the case of the vibration (or perturbation) absence. The first term on the right side of Equation (3) refers to the highly fluctuating signal term due to the Rayleigh backscattering, and the second term is due to the receiver thermal noise. Suppose that a sinusoidal vibration occurs at a specific range point, then the received signal term can be written as:

(4) $$H_{1}:I=v\left(\omega_{p}\right)+I_{\mathrm{RB}}+I_{\mathrm{rec}}$$

which is the alternative hypothesis condition denoted by $H_{1}$. The term **v** is the contribution of a hypothetical unit perturbation with a vibration frequency of $\omega_{p}/2\pi$ (Hz).

When we are concerned with perimeter security and intrusion sensing applications, the frequency range of $\omega_{p}$ is low. In this case, the conventional trace averaging techniques may give satisfactory detection performance to detect such low vibrating events. However, in the case of SHM-related applications, such as crack detection and anomaly detection, the frequency range of the vibrations could be as high as several MHz. Thus, DAS systems with high frequency responses are desired, and we focus on improving the SNR of the DAS system without sacrificing in the frequency bandwidth. In order to highlight the visibility of a particular vibration range bin along the FUT axis (i.e., fast time axis), the following SNR definition is used in this research study:

(5) $${\mathrm{SNR}}_{\left(\mathrm{fast}-\mathrm{time}\right)}=10\log_{10}\left(\frac{\mathrm{the}\mathrm{signal}\mathrm{energy}\mathrm{at}\mathrm{the}\mathrm{vibration}\mathrm{range}\mathrm{bins}}{\mathrm{mean}\mathrm{value}\mathrm{of}\mathrm{the}\mathrm{total}\mathrm{noise}\mathrm{energy}\mathrm{at}\mathrm{other}\mathrm{range}\mathrm{bins}}\right)$$

The result of Equation (5) is expressed in decibels (dB) and will be used as a performance metric in this study. The result is considered to be descriptive of how much speckle noise reduction is achieved.

### 2.2. The Adaptive Matched Filtering Method

Our objective is to reduce the effect of speckle-like noise and detect the presence of such a high frequency vibration and its spatial location by designing an FIR filter. The approach simply ’tunes’ the FIR filter to a signal of interest in such a manner that at the output of the filter, the SNR is maximized. The optimal filtering theory states that if the noise and all undesired components approximate Gaussian processes, the maximization of SNR at the output of the FIR filter is equivalent to a maximization of the probability of detection [17,18].

The filtering process and the scalar output of the proposed FIR filter at the *k*-th range can be written as:

(6) $$z_{k}=\sum_{m=1}^{N}w_{k,n}^{*}i_{k,n}=W_{k}^{H}I_{k}$$

where the superscript * denotes the complex conjugate of a scalar value and H denotes the complex conjugate vector or matrix transpose. The filter weights are collected as:

(7) $$W_{k}={\left[\begin{array}{cccc} w_{1,k} & w_{2,k} & ... & w_{N,k} \end{array}\right]}^{T}$$

We seek a complex valued N-point FIR filter $W_{k}$ such that the output SNR is maximized. The temporally-sampled signal vector (i.e., perturbation signal) of $H_{1}$ can be written as:

(8) $$v_{p}\left(\bar{\omega }\right)=\sigma_{p}s_{k-p}\left(\bar{\omega }\right)$$

(9) $$s_{k-p}\left(\bar{\omega }\right)=\left[\begin{array}{ccccc} 1; & e^{j2\pi \bar{\omega }}; & e^{j4\pi \bar{\omega }}; & ... & e^{\mathrm{jN}\pi \bar{\omega }} \end{array}\right]$$

In the above equation, $\sigma_{p}$ is a random complex voltage, and $\bar{\omega }=f/PRF$ is the normalized sampling frequency. Vector $s_{k-p}\left(\bar{\omega }\right)$ is the set of the signal samples on the slow-time axis with a sampling frequency equal to the PRF. Since our objective is to maximize the SNR at the output of the FIR filter, the SNR on the slow-time axis for a specific range bin can be expressed as:

(10) $${\mathrm{SNR}}_{\left(\mathrm{slow}-\mathrm{time}\right)}=\frac{P_{\mathrm{signal}}}{P_{\left(\mathrm{RB}+\mathrm{rec}\right)}}=\frac{E\{W_{k}^{H}v_{p}\left(\bar{\omega }\right)v_{p}^{H}\left(\bar{\omega }\right)W_{k}\}}{E\{z_{k/H_{0}}z_{k/H_{0}}^{H}\}}$$

(11) $${\mathrm{SNR}}_{\left(\mathrm{slow}-\mathrm{time}\right)}=E\left\{\sigma_{p}^{2}\right\}\frac{W_{k}^{H}ss^{H}W_{k}}{W_{k}^{H}I_{k/H_{0}}I_{k/H_{0}}^{H}W_{k}}=E\left\{\sigma_{p}^{2}\right\}\frac{|W_{k}^{H}s{|}^{2}}{W_{k}^{H}R_{k}W_{k}}$$

where E{.} denotes the expectation operator. $R_{k}$ is the covariance matrix of the noise contributions, and we took $s=s_{k-p}(\bar{\omega )}$ for simplicity in the expressions. By simple mathematical manipulations, the optimum weight vector or the so-called optimal matched filter that maximizes the above equation can be derived as:

(12) $$W_{\mathrm{opt},k}=\alpha R_{k}^{-1}s_{k-p}\left(\bar{\omega }\right)$$

where α is an arbitrary real number. The derivation of the above equation can be found in Appendix A. We can interpret $A=R_{k}^{-1/2}$ as a whitening filter because $Az_{k}z_{k}^{H}A=I$ where I is the identity matrix. Since the whitening filter decorrelates the colored noise input, the FIR filter acts as a correlation canceler. In white noise case, $s_{k-p}\left(\bar{\omega }\right)$ is the matched filter; it has a bandpass response and maximizes SNR. This optimal weight vector can be interpreted as [21]:

(13) $$W_{\mathrm{opt},k}=\alpha \underset{\underset{\text{Filter}}{\mathrm{Whitening}}}{\underset{︸}{\left(R_{k}^{-1/2}\right)}}\underset{\underset{\text{Matched Filter}}{\mathrm{Warped}}}{\underset{︸}{\left(R_{k}^{-1/2}s_{k-p}\left(\bar{\omega }\right)\right)}}$$

where the process in the right-bracket above accommodates the linear transformation applied to the temporal signal vector during the whitening stage. Equations (12) and (13) state that the weights chosen to maximize the SNR are determined via the statistics of background noise, as it was the case in the MMSE approach. This background statistics includes both the speckle-like profile of Rayleigh backscattered data and receiver noise contributions. The only different constant term of the MSNR calculation α is usually set to unity or taken as:

(14) $$\alpha =1/\sqrt{s_{k-p}^{H}\left(\bar{\omega }\right)R_{k}^{-1}s_{k-p}\left(\bar{\omega }\right)}$$

The constant α computed via Equation (14) will normalize noise power at the filter output. In real-world applications, neither the covariance matrix nor the signal vectors are known. For the steering vector computation, if there is no a priori information about the frequency region of interest, the processor has to sweep across the frequency axis to detect the vibration frequencies. For covariance matrix estimation, the situation is quite more complex. For every range bin under interest, the noise covariance is to be estimated from the received samples, and the noise statistics are estimated by the following:

(15) $${\hat{R}}_{k}=\frac{1}{L_{s}}\sum_{l=1}^{L_{s}}I_{k/H_{0}}I_{k/H_{0}}^{H}$$

The vector $I_{k/H_{0}}$ is called the secondary data taken from ranges neighboring to the test range bin under interest, and $L_{s}$ is the number of the neighboring cells. Thus, the weights are computed via Equation (13) with this estimated value of the covariance matrix. The range bin under test is considered as the target range bin containing the primary data. With the estimated noise covariance, our AMF approach computes and applies a distinct complex valued adaptive weight vector for optical pulse returns collected over a specific time interval. This is repeated for every range bin of the ϕ-OTDR trace to detect a vibration at any location (i.e., along the FUT axis). Our detection algorithm can be summarized as follows:

i. The ϕ-OTDR data are collected, and a TPI duration (i.e., size of *N*) is determined.
ii. A training strategy is determined, and the training region along the sensing fiber axis with size $L_{s}$ is selected. Using this training dataset, the covariance matrix of background noise is estimated by Equation (15).
iii. An adaptive weight vector is computed by Equation (13) with the steering vector tuned to the specific vibration frequency of interest.
iv. Using this weight vector, the complex scalar output for the *k*-th range is computed along the sensing fiber by Equation (6), i.e., for all values of k=0,1,…,K−1, and the results are plotted.

In real-world applications with buried fiber optic cables, the noise impact of the operational environment is unknown a priori. For relatively short range applications, such as SHM, the environmental noise may be stationary, and a fixed training region may be used along the whole FUT. However, for long range DAS applications, such as pipeline or border security, the background noise is most likely to change with time. In this case, the estimated covariance needs to be updated according to the specific DAS application.

## 3. Experiments and Results

### 3.1. Methodology

In order to validate the proposed AMF approach with real data, a coherent detection-based ϕ-OTDR hardware has been developed in our laboratory. Both indoor experiments and field tests were conducted with the methodology described below.

**Figure 3:**
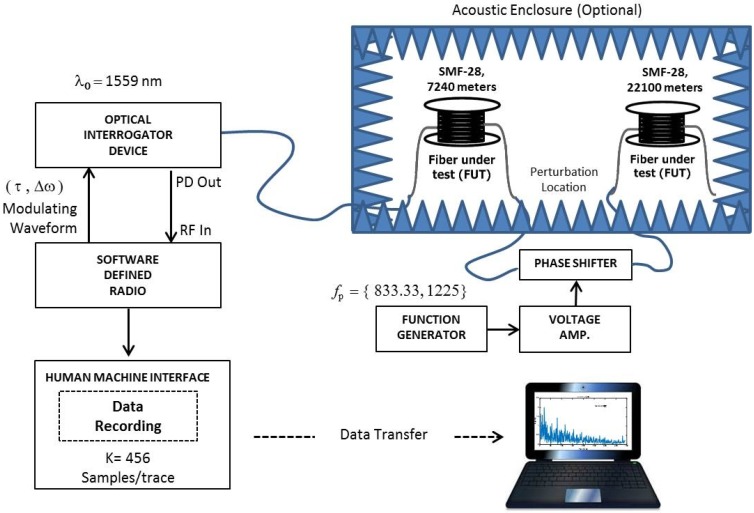
Experimental test setup for the indoor ϕ-OTDR evaluation.

The first phase was dedicated to the comparison of the experimental data with simulated data. The Monte Carlo simulations conducted in our previous study [19] present an ideal situation in which only one test range bin was subject to a synthetic perturbation, while all other simulated range bins contained only Rayleigh backscattered noise without any signal contamination. Since the fiber optic (FO) cables are highly sensitive to acoustic noise in the environment, the fibers on reels were put in an enclosure with specially lined acoustic absorbers to relatively reduce the impact of the environment. The test range bin under interest is extracted outside the enclosure as depicted in Figure 3 below. By this configuration, it was considered that the measured results were directly comparable with the Monte Carlo simulations.

In the second phase of indoor experiments, the acoustic isolation was totally removed, and the AMF approach was tested with the same parameters. Additionally, the intensity of the vibration was reduced to emulate weak signal conditions.

As the final stage, field tests were carried out with the same ϕ-OTDR system by replacing the fiber on reels with a real buried fiber. Real ϕ-OTDR data were gathered at different lateral distances from the buried FO cable by applying various target signals on the ground surface (e.g, walking, hitting with a hammer, digging). The performance of the AMF approach was tested for both strong and weak signal conditions and compared with the conventional trace averaging techniques. After the spectrum of strong vibrations was analyzed to observe the vibration frequencies, the AMF method was applied to weak signals gathered at far distances from the FUT.

### 3.2. Laboratory Experiments

Our experimental setup is shown in Figure 3. The fiber under test (FUT) is a cascaded of two FO cable on reels, which are 7.24 km and 21.1 km long.

As stated above, the cascading point of FO reels was extracted outside the box to induce an acoustic vibration on the 7240-m location. For this purpose, a linear phase-shifter is used to simulate the vibration, and the frequency of the phase shift is adjusted by a function generator (i.e., the frequency of the acoustic vibration is controlled by the function generator). The output of the function generator is amplified to drive the phase shifter due to the given specifications by the vendor.

The experimental system consists of two main 19-inch rack mountable instruments. The first subsystem is called the optical interrogator device (OID) that incorporates all optical and electro-optical components of the DAS system as shown by the dashed lines in the upper left part of Figure 1. The second subsystem is a software-defined radio (SDR)-based receiver, which is used to capture and analyze the ϕ-OTDR signals with digital signal processing (DSP) capabilities. The OID emits optical pulses and receives the backscattered optical power in a cooperative manner with the SDR receiver.

Before the experiments, although no booster optical amplifiers were used at the transmitter stage, the peak optical power launched into the FUT was measured by an optical spectrum analyzer to verify that the system was free of the stimulated Brillouin scattering (SBS) phenomenon, which might have impairments on the received signal [22,23].

The SDR device is responsible for the modulation waveform parameters that are managed by the user. It incorporates a tunable radio frequency (RF) front end tunable up to 3 GHz. The IF and baseband processing section includes high-speed and high-resolution digitization (A/D) and waveform synthesis (D/A) blocks, as well as a field programmable gate array (FPGA) with on-board memory. The SDR serves as the system control unit of the whole system, which is operated via the graphical user interface (GUI) of the SDR receiver. After the electrical signal is processed by the RF front stage of the SDR, it is then digitized by a two-channel 14-bit 250 MSPS A/D and processed by the FPGA block. The baseband I/Q signal is digitally down-converted and decimated for baseband conversion. The raw data are then transferred to another personal computer to be processed in MATLAB. In this research study, the SDR device is only used to capture real-time data and to store it for subsequent processing since real-time implementation in the FPGA is still under development. After decimation, the final number of I/Q data samples recorded by the system for the experiments is 456 samples per trace.

**Figure 4:**
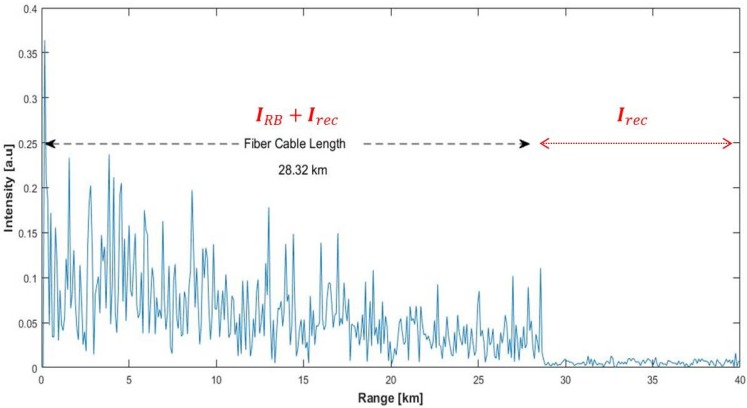
A typical single ϕ-OTDR trace recorded by the laboratory test setup.

The system parameters were set to fixed values before recording the data for different vibration frequencies. The technical specifications and the general system parameters used during experiments are summarized in Table 1. The display range of the GUI was taken as 40 km to observe the total Rayleigh scattering profile easily along the 28.32 km-long FUT. A typical ϕ-OTDR trace gathered from the system is shown in Figure 4. Since the vibration is induced at 7240 m and the resolution is set to 87 m by the system, the target range bin is the 83rd bin via int(7240/87)=83 where int() is the integer operator.

The exponential decaying and speckle-like feature of the Rayleigh backscattering up to the end of the FUT are obvious. The total background noise is composed of the Rayleigh scattering-related part plus the receiver noise term till the end of the FUT, which is 28.32 km long. The data record in the flat region of the plot correspond only to the receiver noise terms.

The AMF approach was tested for several different vibration frequencies, but due to limited space, the results of only two different vibration frequencies are presented here. The information about these sample data records is given in Table 2. The first frequency selected is 833.3 Hz, and it is the same vibration frequency used in our previous simulation study [19] for comparison. The second vibration frequency is 1225 Hz, and it is selected close to the maximum detectable frequency, which is half of the PRF, i.e., 1250 Hz. The vibration frequency $f_{p}=\omega_{p}/2\pi$ is controlled by the function generator and is equal to the frequency of the sinusoidal waveform used to modulate the phase shifter.

**Figure 5:**
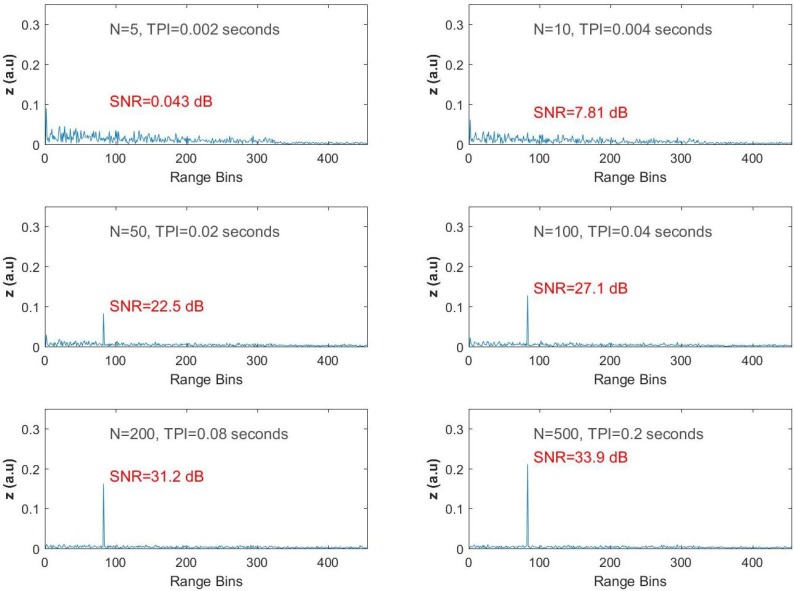
Test results for $\omega_{p}/2\pi$ = 833.33 Hz.

**Figure 6:**
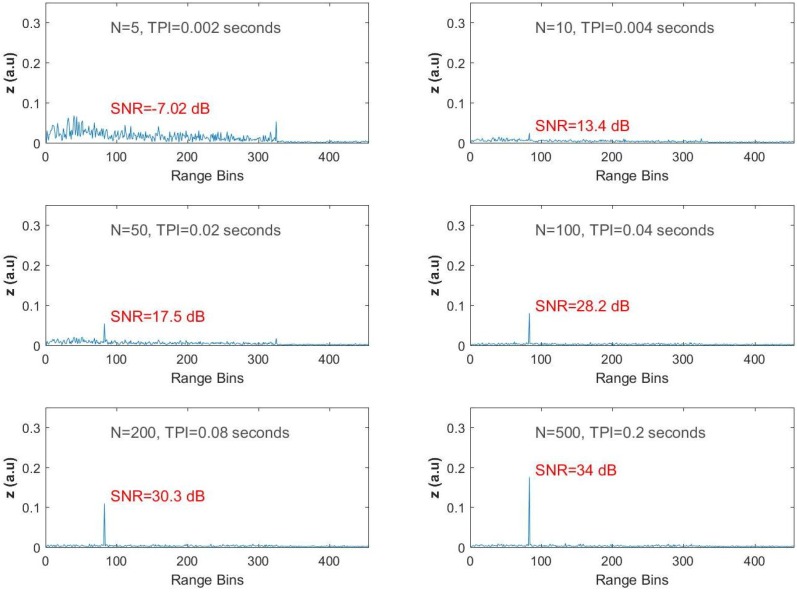
Test results for $\omega_{p}/2\pi$ = 1225 Hz.

After raw ϕ-OTDR data are recorded by the SDR, they are subjected to off-line adaptive processing (i.e., MATLAB routines) with different processing times. The results are summarized in Figure 5 and Figure 6. The algorithm was run several times with FIR filter sizes (i.e., TPI lengths), and the first three range bins on the FUT axis were selected as the training region, i.e., $L_{s}=3$. Taking a fixed region for secondary data is a realistic approach since all of the fiber optic cable reels were in the same environment, which forms a homogeneous background, and there is no need to move this training window on the sensing fiber axis.

In the first experiment, the frequency of the function generator, which drives the phase shifter located at the 83rd bin was set to 833.33 Hz, and data were recorded for about 6.8 s. In the first dataset, the total number of ϕ-OTDR traces was 17,043. The adaptive processing was applied, and filtered ϕ-OTDR traces were obtained for every range bin by setting the tuning frequency of our steering vector equal to the vibration frequency of 833.33 Hz. As mentioned earlier in this text, the performance criterion is to observe the SNR at the output of the filter along the FUT axis. Thus, by remembering Equation (5), the fast-time SNR value after AMF processing is calculated via:

(16) $${\mathrm{SNR}}_{\left(\mathrm{fast}-\mathrm{time}\right)}=10\log10\left(\frac{|z_{\left(k=83\right)}{|}^{2}}{\left(1/(L-1)\right)\sum_{k\neq 83,\forall k}^{}{\left|z_{k}\right|}^{2}}\right)$$

where *L* is the total number of range bins along the FUT, and it is smaller than the total number of range bins recorded. In the data recordings, *L* was computed as 326 according to the total length of the FUT and the spatial resolution, which were 28,340 m and 87 m, respectively (i.e., int (28,340/87) = 326). It is worth noting that all $z_{k}$ values are computed via Equation (6), which inheres the dependency of *N* in the SNR computations.

The computational results of Equation (16) for various values of *N* are displayed in Figure 6, ranging from the top to the bottom and left to the right for increasing *N*, which corresponds to increasing TPI. When the size of the input vector of the FIR filter is equal to five (i.e., N=5 and TPI = 2 ms) there is significant reduction in the speckle like profile of the ϕ-OTDR trace, but no peaks are observed along the range axis, while the calculated SNR is 0.043 dB for the vibration point. Although the SNR is calculated as 7.81 dB when is *N* increased two-fold, no peaks are observed. When N=50 and the resulting TPI duration is increased to 0.02 s, the expected peak at the 83rd range bin is revealed with a resulting SNR value of 22.5 dB.

For the last three trials, the size of the filter is selected as 100, 200 and 500, and the observed SNR values are 27.1 dB, 31.2 dB and 33.9 dB, respectively. As can be easily seen from the filter outputs given in Figure 5, the peak at the 83rd range bin becomes obvious, while the background noise is severely suppressed with increasing *N*.

The same steps were repeated for the second dataset recorded when $f_{p}$ = 1225 Hz and with the test frequency equal to 1225 Hz. For N=5, the SNR is −7.02 dB, and there is not enough reduction in the background noise to reveal the vibration signal. A high reflection is observed at the end of the FO cable. However, when the size of the FIR filter is increased to 10, the peak is slightly clear at the 83rd bin. For N=50, the peak is observed with an SNR value of 17.5 dB. As *N* is increased to 100, 200 and 500, the results are similar to the ones obtained for the previous data with the fast-time SNR values of 28.2 dB, 30.3 dB and 34 dB, respectively, as shown in Figure 6.

**Figure 7:**
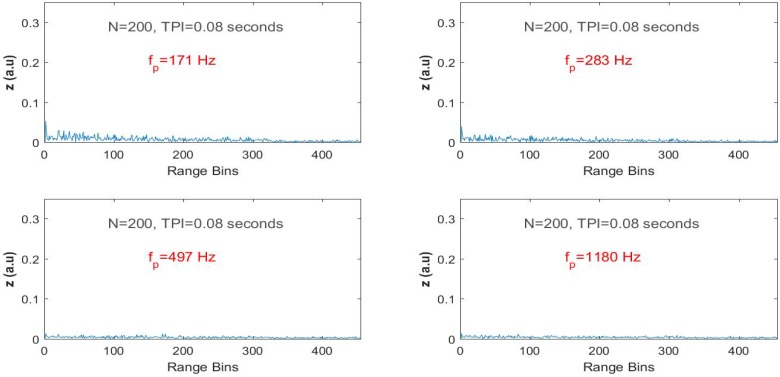
Indoor test results with the steering vector tuned to non-vibrating frequencies.

For the second dataset, the filter outputs were tested for some arbitrary frequencies that are not equal to the vibration frequency such as 171 Hz, 283 Hz, 497 Hz and 1180 Hz. It can be seen in Figure 7 that none of these tuned frequencies yielded spikes at the output of the FIR filter. As the algorithm seeks vibrations over all range bins, the filter output is only maximized when the test frequency matches the real vibration.

The measurement results were compared with the results of the previous simulation study [19]. In that previous research, Monte Carlo simulations were carried out with the same system parameters of the experimental setup. The simulation study was based on the theoretical model given in [8].

**Figure 8:**
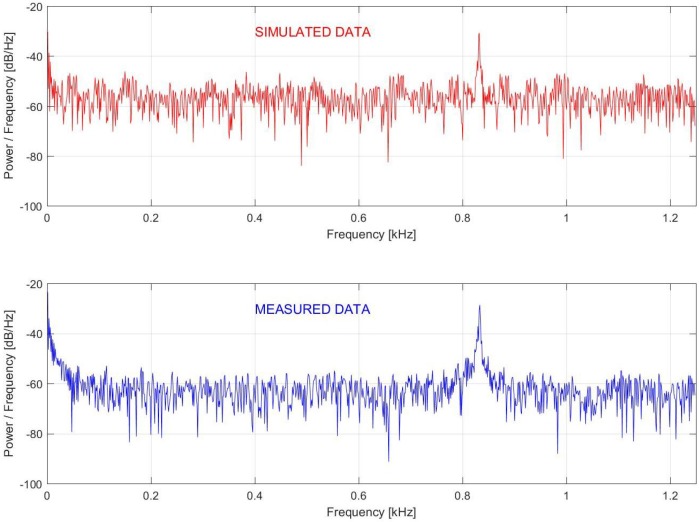
Power spectrum density of the simulated and measured vibration of 833.33 Hz at the vibration location.

For this comparison, the fast Fourier transform (FFT)-based power spectrum of the 83rd bin with 1000 consecutive ϕ-OTDR traces was used. As can be seen in Figure 8, both simulated and measured data yielded a peak at 0.83 kHz, which is equal to the vibration frequency.

**Figure 9:**
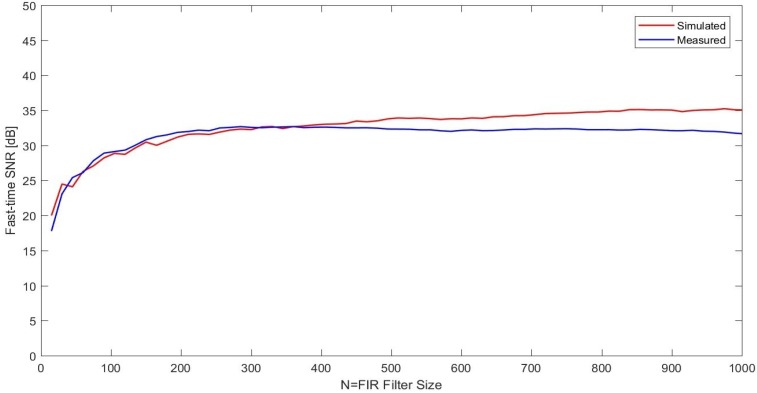
SNR versus *N* curve for the simulated and measured indoor ϕ-OTDR data.

After observing the periodograms, the AMF algorithm was executed for both measured and simulated datasets for increasing *N*. The SNR versus *N* plots are given in Figure 9. It can be concluded in general that the simulation results are in good agreement with the experimental results. The main difference seems to be the relative asymptotic behavior in the measurement data compared to the simulation results.

**Figure 10:**
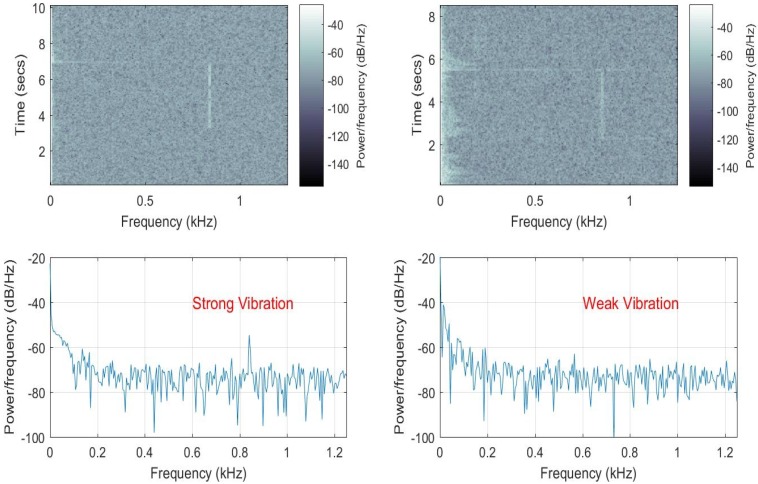
Power spectrum density plots for the last two datasets at the target range bin. The upper row is the time-frequency plot for the whole recording while the graphs in the lower designate the power spectral densities of the strong and weak vibration signals with $f_{p}=833.33$ Hz.

For the second phase of the indoor experiments, the AMF method was tested with the acoustic enclosure removed. Two different vibration datasets were recorded with the 833-Hz vibration turned on for approximately 3 s to emulate “strong” and “weak” non-continuous vibrations. For the strong vibration case, the amplitude of the signal generator was adjusted to a level that the spectrum of the signal exhibits a visible peak. For the weak vibration case, the amplitude was decreased to a level where the peak is obscured by the noise spectrum. The spectrogram plots computed in MATLAB are presented in the upper row of Figure 10. The periodograms were computed for only the signal durations to verify the visibility of the signals. As shown in the lower row of Figure 10, the SNR in the strong signal case is about 15 dB over the noise floor. From the graph presented in the lower right part of the figure, it is impossible to decide on the presence of a vibrating signal with the power spectrum.

**Figure 11:**
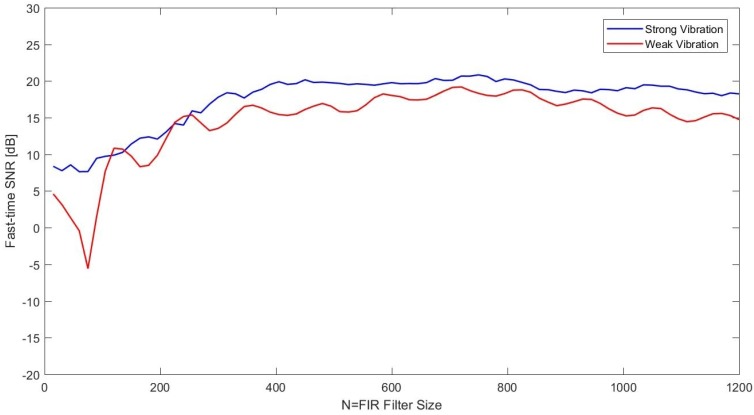
SNR versus *N* curve for the strong and weak vibration ϕ-OTDR data with $f_{p}=833.33$ Hz.

The fast-time SNR values versus FIR size were computed for both strong and weak signal conditions. It can be seen from Figure 11 that the trend of the SNR curve is similar to those obtained for the measured data with acoustic isolation. There is a negative offset in the SNR axis, which can be expected due to the reduced power of vibration signal. The only interesting point observed was the oscillating behavior of the SNR curve for the weak signal test.

**Figure 12:**
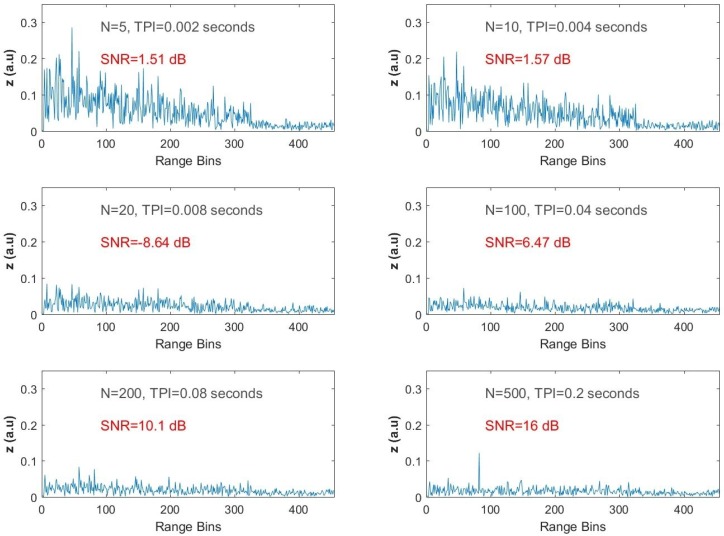
Indoor test results for the weak vibration of 833.33 Hz with acoustic isolation removed.

The AMF output was tested for various FIR sizes for the weak vibration. Figure 12 presents the output intensity profiles obtained for the same set of *N* values as previously selected. For the weak vibration, the peak at the 83rd range bin is not observed unless *N* = 200, but it is not very dominant since the next peak on the left seems to be another candidate, which may cause a false alarm. The significant noise reduction is obtained, and the vibration is clearly emphasized when *N* = 500.

### 3.3. Field Tests

**Figure 13:**
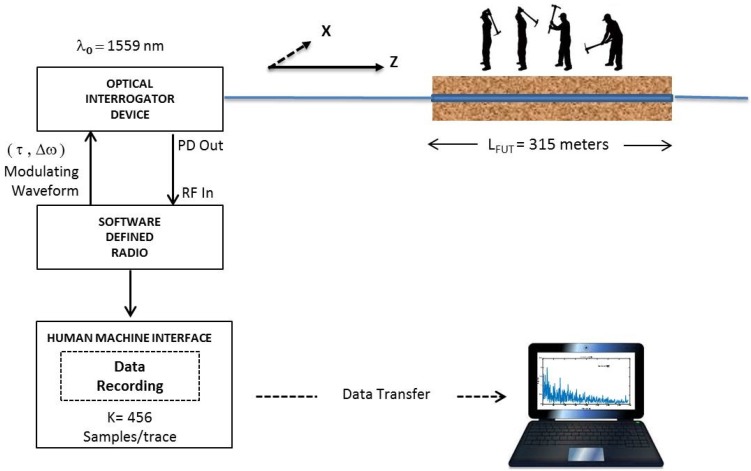
Experimental ϕ-OTDR test setup for field tests.

**Figure 14:**
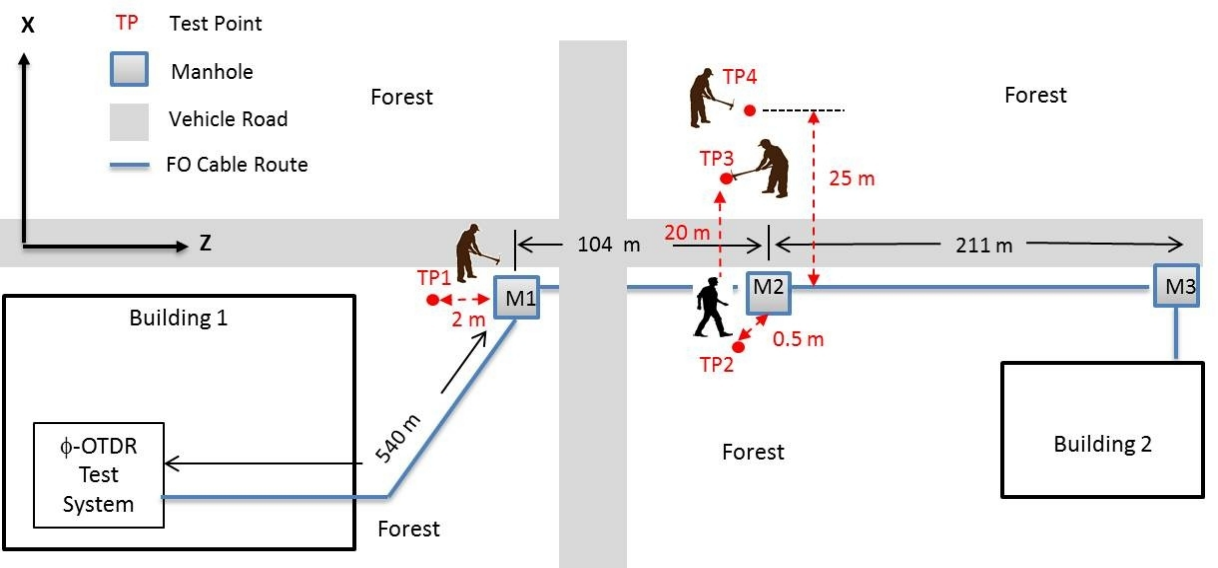
Generic schematic of the outdoor test points.

**Figure 15:**
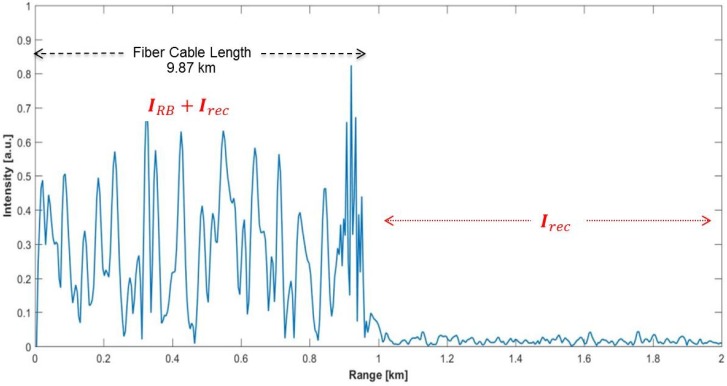
A typical ϕ-OTDR trace recorded during field tests with the buried fiber.

To validate the proposed approach in real-world conditions, field tests with a real fiber optic bundle buried approximately 0.5 m underground were carried out on the TÜBİTAK (The Scientific and Technological Research Council of Turkey) campus site located in Gebze. The acoustic enclosure and the fiber on reels were removed from the experimental setup, and the ϕ-OTDR system was connected to the buried fiber through a patch panel in the office area as depicted in Figure 13. The route of this selected intranet line between two facilities is roughly illustrated in Figure 14. The FO bundle consists of 12 different thick coated SMF cables that were installed with a protection sheath made of steel. One spare fiber cable was selected as the FUT to observe the sensitivity of this telecommunication line for a potential DAS application. The exact length of the FUT was estimated as 987 m via our ϕ-OTDR system screen as shown in Figure 15. Before the field tests, several manholes were inspected to verify the route and to assign the correct range bin locations on the observed OTDR traces. The starting and ending range of the effective buried length was estimated as 540 and 855 m, respectively, by perturbing the manhole covers with footsteps. Several tests were conducted between the dates of 19 April 2017 and 3 May 2017 with this buried FUT, and real ϕ-OTDR data were recorded during two weeks at different locations and with varying distances from the FUT. Due to the limited scope, only the results of the four data summarized in Table 3 are presented in this paper.

Since the effective test length of the FUT was only about 315 m, by adjusting the OID and SDR parameters, the spatial resolution was increased to 25 m by reducing the laser pulse width to 244 ns. Oversampling was performed for better visualization of the results on the range gates of interest. Due to the on-board storage limitations of the DSP hardware, the recorded range was fixed to 2000 m with 456 samples per every OTDR trace independently from the adjusted PRF. This synthetic enhancement of the SR with $\Delta z_{s}$ = 2000/456 = 4.39 m is the result of the oversampling, and it is higher than the actual SR of the system. All other system parameters were kept the same as before. The modified system parameters used during field tests are summarized in Table 4.

**Figure 16:**
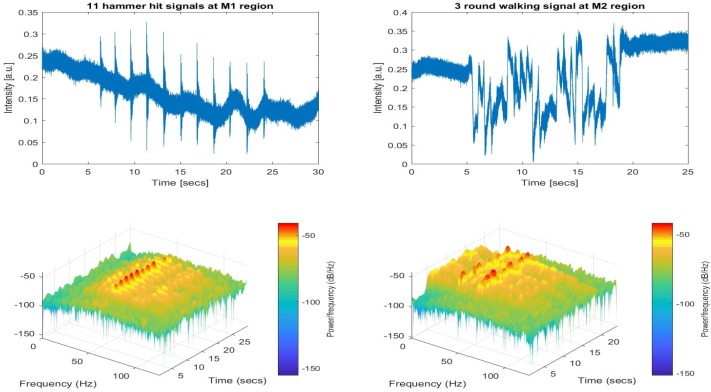
Two sample ϕ-OTDR intensity data recorded near Manholes 1 (TP1) and 2 (TP2).

The first two field datasets were recorded with strong perturbations to observe the spectrum of the vibrations in close vicinity of the buried cable. The data shown in left column of Figure 16 refer to the intensities of the signal recorded at the first test point (TP1), while the gravel path 2 m away from the manhole was hit with a big hammer.

As can be seen from the intensity profile in the graph, the total of 11 sequential hits is considerably strong and easily “heard” by the FUT in the 123rd range bin. The time-frequency analysis shows that most of the signal energy is concentrated in the 20 Hz–100 Hz region giving a dominant peak around 50 Hz. The second field dataset was recorded while a 90-kg man circled the second manhole three times with uniform footsteps as illustrated on the right side of Figure 16. Similarly, the presence of a signal component is obvious both in the time domain and the frequency domain plots. Two dominant peaks were observed around 30 Hz and 45 Hz, as can be seen from the spectrogram processing of the footsteps.

**Figure 17:**
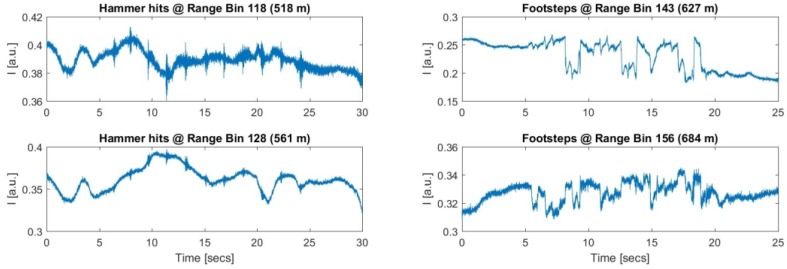
Recorded intensities at different range bins for the two test points TP1 and TP2.

The analysis of the raw ϕ-OTDR data for each range bin revealed that the induced acoustic energy on the FUT was spread on several range bins. The strongest signals recorded for the first and the second manholes were observed on 123rd and 147th range bins, respectively. The distance between the two range bins were computed as (147 − 123)$\times \Delta z_{s}$ = 105.3 m, which was consistent with the measured distance of 104 m, as depicted in Figure 14. The range bins with the weakest contributions were noted along the FUT for every test during the experiments. To give more insight about the amount of energy coupled to the neighboring range bins, the range bin recordings with the least visible signals are plotted in Figure 17. All of the hammer hits are weakly heard by the 118th and 128th range bins. Thus, the vibrations were spread over a range of (128 − 118 )$\times \Delta z_{s}$ = 43.9 m. For the walking experiment conducted at TP2, the footstep signals are visible along a range of 684 m − 627 m = 57 m, as can be seen from the right side of the Figure 17, with the corresponding range bins of 156 and 143.

**Figure 18:**
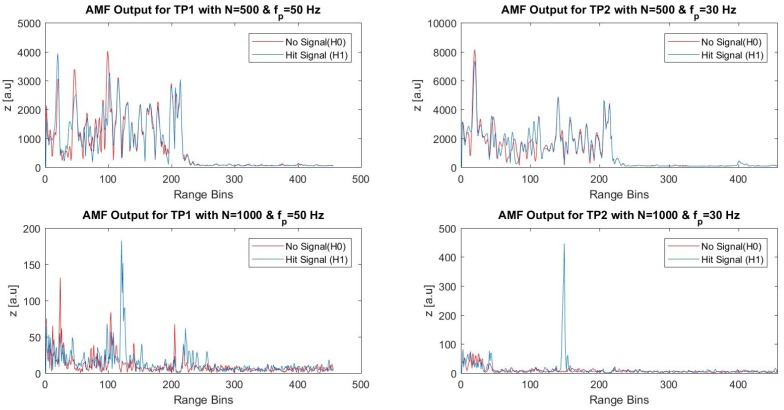
Adaptive temporal filtering (AMF) outputs for TP1 ($f_{p}=50$ Hz) and TP2 ($f_{p}=30$ Hz) with *N* = 500 and *N* = 1000.

The field data were subject to the AMF approach with similar steps followed before. The vibration signals recorded during field tests were no longer pure continuous sinusoidal signals. In order to analyze the “noise only” and “signal presence” cases with the AMF approach, the start of and the end of the durations of each signal shot were noted, and the AMF method was tested with the observed peak frequencies. For the fifth dataset, the first 400 ms of the record and the 400 ms duration of the sixth hammer hit were selected as inputs to the FIR filter in order to analyze the tested noise only (H0) and signal presence (H1) conditions. Since the dominant vibration was observed at 50 Hz for this dataset, the AMF method was tested with the FIR tuned to 50 Hz. From the results shown in Figure 18, the speckle profile was still dominant, although the FIR size was set to *N* = 500. It has been observed after several trials that significant noise suppression was obtained when *N* reaches 1000. Almost similar results were observed with the sixth dataset when the FIR was tuned 30 Hz, as shown in the right column of Figure 18. All of the range bins along the FUT were tested with the same FIR size and the test frequencies. The maxima of the FIR output were observed at the correct range bins as shown by the blue lines: the peak for the fifth dataset was observed around the 123rd range bin, and the peak for the sixth dataset was obtained around the 147th range bin as expected. The FIR outputs responded with no peaks for the “noise only” cases, which are shown by red lines in the lower row of Figure 18.

The last step was dedicated to the testing of the AMF method for weak signal conditions. In order to meet the noisy case, the test distances from the FUT were increased step by step where the acoustic vibrations on the relevant range bins became hardly seen by the operator on the system monitor. The system parameters were set to display the differences of averaged measured ϕ-OTDR traces with an averaging number ranging from 10 to 100, which was adjustable in real time by the operator. After the desired conditions were met, the tests were repeated to record the raw data without any averaging or any other preprocessing.

**Figure 19:**
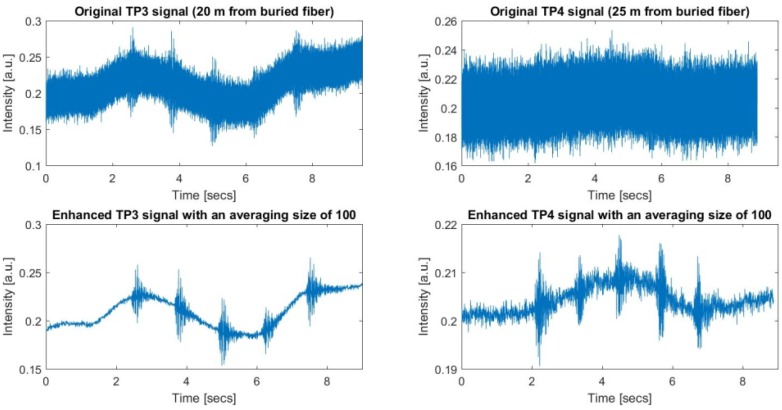
Raw and enhanced ϕ-OTDR data at the 147th range bin for TP3 and TP4.

The recorded raw data shown in Figure 19 below were gathered seven days after the above presented recordings in almost similar environmental conditions. The first data shown in the left side of the figure are the recording of five sequential hammer hits at a lateral distance of 20 m from the second manhole (seventh dataset), while the second data shown on the right side of the figure are the recording of another five sequential hammer hits gathered at a lateral distance of 25 m from the same manhole (eighth dataset).

The upper row presents the original record, while the lower row shows the processed versions of the same data with an average size of 100. As can be seen in the upper right plot, the hammer hits recorded at 25 m were highly obscured by noise that can be considered as a very weak signal condition since these signals became visible only when sufficient averaging was applied. Before applying the whole data record to the FIR filtering with appropriately selected FIR sizes and the vibration frequency, it was considered first to analyze the fast-time SNR values for every signal. As the start and the durations were noted on the enhanced signal, these time indices and duration values were used to test the output of the AMF for all H1 conditions. In order to compare the AMF with the conventional averaging techniques, the fast-time SNR was used as a figure of merit as mentioned before. The signal range bin set for $z_{k}$ was collected with k∈ { 138,...,156 }, while all other range bins along the “effective” FUT axis were taken for computation of the noise energy. Thus, the noise range bin set is the union of neighboring regions {110,...,137} and {157,...,180}.

**Figure 20:**
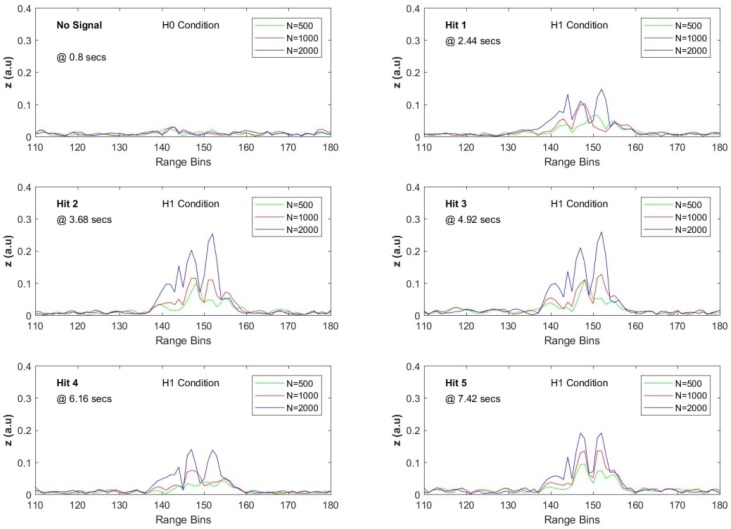
SNR values along the effective FUT axis with three different *N* for the seventh dataset.

The noise only condition and the five different signal presence conditions were tested with three different FIR sizes when the steering vector was tuned to 50 Hz. The results obtained for the seventh dataset are given in Figure 20, and the comparison of SNR values with the SAM is listed in Table 5. The original raw data were processed with three different FIR sizes as 500, 1000 and 2000. The SAM method was applied with various pairs of (M,L) values, (i.e., SAM(M,L)) where *M* denotes the averaging block size and *L* is the lag value used in sequentially differentiating the ϕ-OTDR traces. After several trials with the 7th dataset, the relatively high SNR results obtained via SAM(100,50) and SAM(200,15) were selected for comparison, which are given in Table 5. For the proposed AMF method, the results show that significant SNR improvement is achieved with all selected FIR sizes, but a clear superiority to SAM(M,L) is achieved when *N* is increased over 1000.

**Figure 21:**
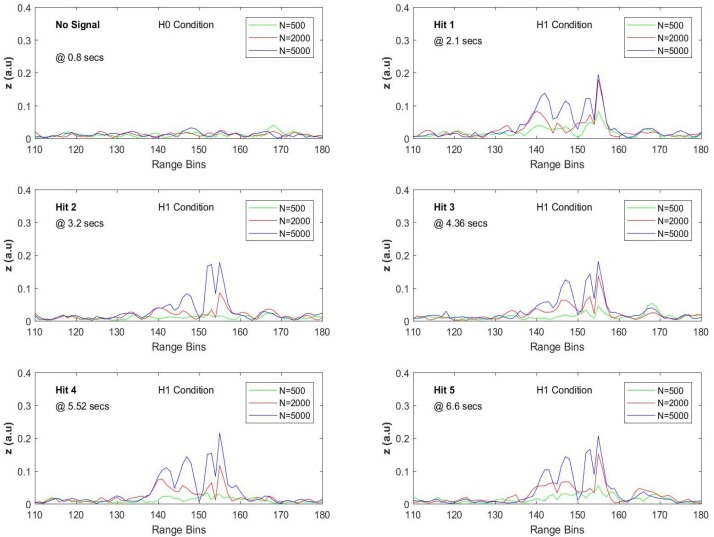
SNR values along the effective FUT axis with three different *N* for the eighth dataset.

The AMF outputs and the SNR values computed for the last data are shown in Figure 21 and listed in Table 6, respectively. For the SAM(M,L) method, after several trials with various average size and lag values, it was observed that the best SNR performance was achieved with SAM(200,15). The noise contamination for these data is more severe compared to the previous dataset, and significant noise reduction performance is achieved when *N* is selected over 2000 for the AMF method. More than 10 dB of SNR values can be easily obtained by increasing the FIR size over 3000 with penalties in processing times.

### 3.4. Discussion

For the data gathered indoors with the acoustic isolation, it can be easily deduced from the results that the vibration is not solely observable unless the *N*, the FIR vector size (or in other words, the number of consecutive ϕ-OTDR traces processed at the same time), is over 50. When *N* is increased to values higher than 100, the amount of speckle reduction is significant, which means that the $I_{\mathrm{RB}}$ term of the background noise is highly suppressed. When the acoustic isolation is removed and the vibration intensity is reduced to lower levels, more than a 10-dB speckle reduction can only be achieved with a much higher value of *N*. This is expected since both simulation data and the measurement data with isolation present the ideal situation. When the isolation is removed, the whole FUT is subject to more interference, and the vibration signal is highly obscured by the increased amplitude fluctuations of the ϕ-OTDR traces.

All field tests yielded the same results, and the vibration energy is better visualized when the FIR size is increased. We can say that the longer the AMF processing interval, the better the speckle reduction is. It is very clear from the test results that an FIR size of at least 1000 seems to be mandatory to reach a minimum SNR level of 10 dB for weak signal detection. On the other hand, when we are only concerned with the real measurement data, the processing time and computational load will increase with increasing *N*; it will not be more beneficial to use very large values of *N* for real-time applications. It is worth emphasizing that we did not take any care to avoid SOP changes in our setup; thus, fast-time SNR analysis for longer durations may exhibit decreasing behavior, which is another research topic for us.

**Figure 22:**
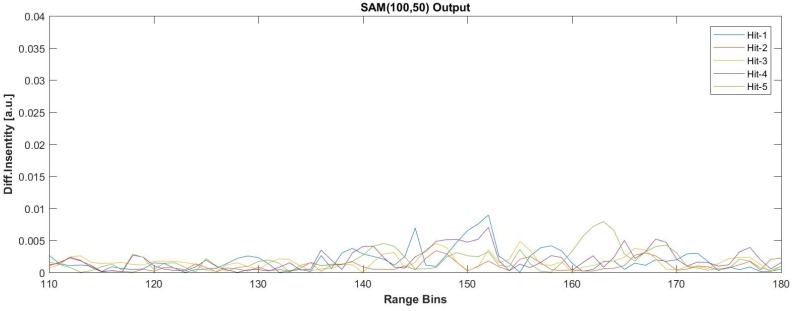
SAM(100,50) processing results for the five hammer hit durations of the eighth dataset.

**Figure 23:**
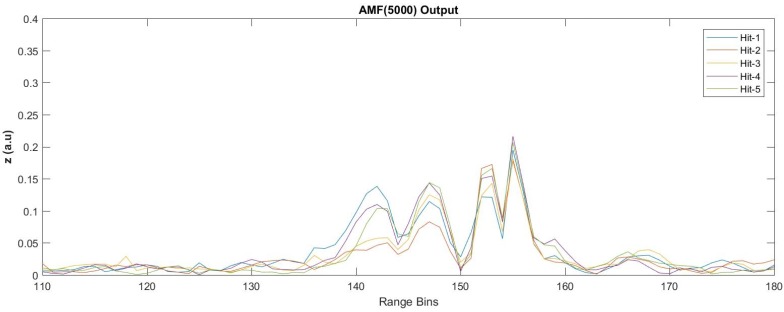
AMF(5000) processing results for the five hammer hit durations of the eighth dataset when the FIR is tuned to 50 Hz.

Besides the computed fast-time SNR values given in Table 5 and Table 6, the performance of the proposed AMF approach in comparison with the SAM(M,L) method for the weak vibrating signal condition can be better observed with the two sample results given below. Figure 22 presents the performance of the SAM (100,50) method for the detection of all five hammer hits in the eighth dataset. It can be easily seen from Figure 22 that the differentiated intensity profile obtained from SAM(100,50) does not provide clear visibility for the hitting activities. Figure 23 is the processed version of the same dataset with the AMF approach with an FIR size of 5000 and $f_{p}=50$ Hz. As the noise background is highly suppressed, the acoustic vibrations distributed along the FUT axis are better visualized, and all of the five hammer hits designated by different colors in Figure 23 are clearly detectable. The training region size selected during AMF(5000) processing was 20 (i.e., $L_{s}\in \left[231,250\right]$).

The main advantage of the AMF approach is that there is no need for trace averaging, which results in the reduction of the system bandwidth. During the field tests of weak vibrations, the PRF was set to 25 kHz, and the MDF was naturally 12.5 kHz. As an example, in order to achieve an SNR improvement of 10 dB in this scheme, then we would need to coherently sum up 100 consecutive ϕ-OTDR traces and use the average of them. This would reduce the MDF down to 12,500/100 = 125 Hz. If the vibration of interest is higher than this value, it will be impossible to detect the frequency of vibration. Thus, averaging can only enhance the SNR in terms of the spatial localization of the vibrations. In our approach, more than 10 dB of SNR improvement are achievable without any impact on the frequency response of the DAS system, and increasing the size of the FIR does not affect the system frequency response.

As a conclusion, the proposed AMF approach is superior to all conventional averaging techniques due to its capability of detecting the location and frequency at the same time while preserving the frequency response of the system. Additionally, no frequency domain analysis is required to threshold the noise components as applied in several transform domain-based techniques, such as wavelet denoising. Lastly, the achieved SNR values with large FIR filter sizes are higher than all of the reported SNR values in the literature [9,10,11,12,13,14,15].

The main processing requirement of the AMF method is the need for a brute search for all possible vibrations on the frequency axis. This is not difficult with today’s technology and thanks to the high computational power brought by parallel processing capability of FPGAs and multi-core processors, such as graphics processor units (GPU). The AMF method was implemented in NVIDIA compute unified device architecture (CUDA) for using this parallel computation power of commercial of the shelf (COTS) GPUs. It has been observed that searching for all frequencies with 1-Hz resolution with *N* less than 500 for all 456 range bins took less than a second. During these tests, the computation of the inverse of the covariance matrix was implemented in the central processing unit (CPU), while the other necessary tasks were computed by the GPU. The platform used was a desktop computer with Intel Core^TM^ i7-3770K CPU (3.5 GHz CPU with 32 GB RAM) and a GeForce GTX 680 GPU having 512 CUDA cores.

We believe that the AMF is applicable not only for coherent detection-based ϕ-OTDR systems, but will be successfully applied for direct detection-based systems, as well. The underlying theory for the success of the AMF technique is that it whitens the backscattered data and applies the well-known matched filtering theory to the data. The filter is actually matched to the vibration when the tuned frequency is the vibration frequency. Since the optical Rayleigh backscattered traces are inherently correlated, the correlation canceling property of the proposed AMF is exploited very efficiently in this detection scheme. Thus, regardless of the optical detection employed in the interrogation unit, the AMF method can be applied efficiently for all types of Rayleigh scattered optical data. The validation of the AMF method for direct detection systems will be one of our next research topics.

## 4. Conclusions and Future Work

An adaptive temporal match filtering of the ϕ-OTDR signals was reported in this study, to reduce the effect of background noise for improved vibration detection. Due to the inherent temporal correlation between the ϕ-OTDR traces, it was expected to observe significant suppression of the background noise by pre-whitening of the temporal traces. The temporal adaptive processing algorithms were tested on real experimental data including field tests. The results showed that without introducing any optical amplification in the architecture and without any digital preprocessing of the backscattered data, the standalone adaptive processing of ϕ-OTDR traces achieved significant SNR improvements. This time-domain technique is capable of detecting weak vibrations by increasing the filter size without any impact on the system frequency response. The weaker the signal to be detected, the larger the size of the filter required for significant noise reduction. The penalties paid due to the increase in processing time for larger filter sizes can be easily compensated by parallel processing algorithms. Thus, we believe that adaptive FIR processing is a promising technique as a vibration sensor for both SHM and intrusion sensing applications.

Further experiments with the direct detection scheme will be conducted as the next step. Our future work will be focused on practical real-time implementations of the adaptive FIR processing in DAS systems for industrial applications with buried fiber optic cables.

## Figures and Tables

**Table 1:** Test setup and systems parameters. AOM, acousto-optic modulator; FUT, fiber under test; PRF, pulse repetition frequency.

System Component	Value
Laser Wavelength	1559 nm
Laser Source Power	22 mW
Laser Linewidth	<3 kHz
OLOPower	2.2 mW
AOM Frequency	100 MHz
NEP of the Balanced Detector	6 pW/Hz
Type of FUT	Corning^®^ SMF-28^®^
FO refractive Index (n)	1.467
Interrogating PRF	2500 Hz
Pulse Width	860 ns
Spatial Resolution (Δz)	87 m
Total Optical Insertion Loss	6 dB
Length of FUT	28,340 m
Number of Range Bins/Trace	456
Vibration Location	7240 m
Range Bin of Vibration	83

**Table 2:** Indoor test setup and system parameters.

Record	Frames	Duration	Vibration	Acoustic	Vibration
Filename	Recorded	(seconds)	Frequency (Hz)	Isolation	Intensity
Dataset-1	17,043	6.82	833.33	Yes	Strong
Dataset-2	13,753	5.44	1225	Yes	Strong
Dataset-3	25,588	10.23	833.33	No	Strong
Dataset-4	21,499	8.60	833.33	No	Weak

**Table 3:** Summary of the test signals used during field tests.

Record	Frames	Signal	Type of	Number of	Lateral Distance
Filename	Recorded	Duration (s)	Target Signal	Hits/Duration	from the FUT (m)
Dataset-5	309,930	30.99	Hammer Hit	11	2
Dataset-6	251,005	25.10	Walking	3 s	0.5
Dataset-7	245,005	9.80	Hammer Hit	5	20
Dataset-8	222,030	8.88	Hammer Hit	5	25

**Table 4:** Modified system parameters for field tests. TP, test point.

System Component	Value
Interrogating PRF	10 kHz (Datasets-5,-6), 25 kHz (Datasets-7,-8)
Pulse Width	244 ns
Actual Spatial Resolution ($\Delta z_{a}$)	25 m
Synthetic Spatial Resolution ($\Delta z_{s}$)	4.39 m
Length of FUT	987 m
Number of Range Bins/Trace	456
Vibration Location Centers	541 m for TP1, 655 m for TP2, TP3 and TP4
Range Bin(s) of Vibration Regions	[118,128] for TP1, [138,156] for TP2, TP3 and TP4

**Table 5:** SNR results with $f_{p}=50$ Hz at TP3 (lateral distance from FUT: 20 m). SAM, separate averaging method.

Method	Hit-1	Hit-2	Hit-3	Hit-4	Hit-5
SAM (100, 50)	9.52	10.39	11.69	8.82	17.71
SAM (200, 15)	12.79	7.22	8.71	10.19	12.40
AMF (500)	11.52	13.17	12.26	10.77	12.27
AMF (1000)	12.12	15.53	14.65	13.59	15.21
AMF (2000)	17.75	23.22	20.77	18.47	18.99

**Table 6:** SNR results with $f_{p}=50$ Hz at TP4 (lateral distance from FUT: 25 m).

Method	Hit-1	Hit-2	Hit-3	Hit-4	Hit-5
SAM (100, 50)	8.13	2.79	4.47	4.55	0.35
SAM (200, 15)	9.72	4.47	10.70	10.50	7.35
AMF (500)	8.17	0.40	1.17	5.61	5.91
AMF (1000)	11.81	5.10	5.98	8.37	6.18
AMF (2000)	11.81	6.36	11.55	12.42	10.69
AMF (3000)	13.36	10.10	12.17	13.64	12.41
AMF (5000)	14.77	14.35	13.94	15.45	15.93

## Appendix A. Derivation of the Maximum SNR Equation

Consider the SNR definition given by Equation (12). For simplicity, we will assume a unit amplitude perturbation signal (i.e., $\sigma_{p}=1$), and with some matrix manipulations and exploring the Hermitian symmetry of the covariance matrix, we can write:

(A1) $${\mathrm{SNR}}_{\left(\mathrm{slow}-\mathrm{time}\right)}=\frac{|{\left(R_{k}^{1/2}W_{k}\right)}^{H}\left(R_{k}^{-1/2}s\right){|}^{2}}{{\left(R_{k}^{1/2}W_{k}\right)}^{H}\left(R_{k}^{1/2}W_{k}\right)}$$

To find an upper bound for the above expression, we can use the well-known Cauchy–Schwarz inequality and write:

(A2) $${\mathrm{SNR}}_{\left(\mathrm{slow}-\mathrm{time}\right)}=\frac{|{\left(R_{k}^{1/2}W_{k}\right)}^{H}\left(R_{k}^{-1/2}s\right){|}^{2}}{{\left(R_{k}^{1/2}W_{k}\right)}^{H}\left(R_{k}^{1/2}W_{k}\right)}\leq \frac{\left[{\left(R_{k}^{1/2}W_{k}\right)}^{H}\left(R_{k}^{1/2}W_{k}\right)\right]\left[{\left(R_{k}^{-1/2}s\right)}^{H}\left(R_{k}^{-1/2}s\right)\right]}{{\left(R_{k}^{1/2}W_{k}\right)}^{H}\left(R_{k}^{1/2}W_{k}\right)}$$

Thus, the above equation can be simplified as:

(A3) $${\mathrm{SNR}}_{\left(\mathrm{slow}-\mathrm{time}\right)}=\frac{|{\left(R_{k}^{1/2}W_{k}\right)}^{H}\left(R_{k}^{-1/2}s\right){|}^{2}}{{\left(R_{k}^{1/2}W_{k}\right)}^{H}\left(R_{k}^{1/2}W_{k}\right)}\leq s^{H}R_{k}^{-1}s$$

We can achieve the upper bound if we choose:

(A4) $$R_{k}^{1/2}W_{k}=\alpha R_{k}^{-1/2}s$$

Here, α is an arbitrary real number. We can verify this upper bound condition by plugging it in the Equation (A4) and obtain:

(A5) $${\mathrm{SNR}}_{(\mathrm{slow}-\mathrm{time}),\mathrm{MAX}}=\frac{|s^{H}R_{k}^{-1}{s|}^{2}}{s^{H}R_{k}^{-1}s}=s^{H}R_{k}^{-1}s$$

Thus, the optimal matched filter is:

(A6) $$W_{\mathrm{opt},k}=\alpha R_{k}^{-1}s$$
